# Sustainability of Donor-Funded Health-Related Programs Beyond the Funding Lifecycle in Africa: A Systematic Review

**DOI:** 10.7759/cureus.24643

**Published:** 2022-05-01

**Authors:** Olayinka S Ilesanmi, Aanuoluwapo A Afolabi

**Affiliations:** 1 Department of Community Medicine, University of Ibadan, Ibadan, NGA; 2 Department of Medicine, University of Ibadan, Ibadan, NGA

**Keywords:** africa, sustainability, donor funding, health projects, health interventions

## Abstract

This study aimed to determine if implemented donor-funded health related-programs in Africa were sustained beyond the funding lifecycle and determine their facilitators and impeders. A systematic review was conducted after the documentation of a study protocol. A database search was done across three databases namely Google Scholar, PubMed, and Medline between January 27 and February 15, 2022. All peer-reviewed articles on sustainability of health interventions in Africa published between 2015 and 2021 that provided one or more context-relevant definitions of sustainability were included. Publications with no use of quantitative or qualitative methods and studies with no information on project evaluation after initial implementation were excluded. Screening of titles and abstracts was done, and the full texts of all relevant articles were retrieved. The risk of bias in systematic reviews (ROBIS) tool was used to assess the risk of bias in the systematic review. Overall, 4,876 articles were retrieved, and only nine articles were eligible for inclusion in the review following the removal of duplicates. Overall, sustainability was described in only three of the five regions in Africa. Donor-funded projects were sustained beyond the funding lifecycle in seven (77.8%) studies. Facilitators of sustainability in Africa included community ownership of the project through the engagement of community stakeholders in the design and implementation of such projects, use of locally available resources, sound infrastructure, and the constitution of interdisciplinary team to facilitate capacity building. Impeders to the sustainability of donor-funded projects included weak health systems exemplified in poor documentation and integration of records, lack of financial leadership, shortage of resources, political interference, poor feedback mechanism, and weak donor-community interactions. From the ROBIS tool, a low risk of bias existed in the studies included in the review. Although the included studies appropriately considered the review’s research question, seven studies had a low risk of bias in the domains one to three, and two studies had high risk of bias in domain four. To derive maximum benefits from donor-funded health interventions, sustainability of such projects is key. During program planning phase, context-based facilitators of sustainability should be promoted, while impeders are immediately addressed.

## Introduction and background

The availability of donor-funded health and health-related projects has led to the restoration, improvement, and maintenance of health [1]. Many programs in maternal health, reproductive health, disease prevention, as well as mental health projects have been implemented [2-4]. Public health programs focused on health improvement have been shown to deliver positive health outcomes, however, the maintenance of these programs over long periods and beyond the funding lifecycle has often been challenging [5]. The provision of financial resources from a particular funder only lasts for a defined period, after which funding is expected to be received from other sources [1]. Over time, the sustainability of a program is influenced by various elements, such as the content of program activities, partnerships at community level, organizational practices, and perceived benefits of the program [6]. These elements termed sustainability outcomes reflect the sustained continuation of a program to meet its intended outcomes.

The United States Agency for International Development defines sustainability in a more focused manner as “the capacity to maintain program services at a level that will provide ongoing prevention and treatment for a health problem after termination of major financial, managerial, and technical assistance from an external donor” [7]. In their study, Scheirer and Dearing defined sustainability as “the continued use of program components and activities for the continued achievement of desirable program and population outcomes” [8]. This definition was adapted by Schell et al., who highlighted the importance of the element of time in knowing the true definition of sustainability [9]. There, sustainability was defined as the ability to maintain programming and its benefits over time [9]. In another context, however, the timeline of a donor-funded program has been described as an insufficient and inaccurate estimate to measure the sustainability of an intervention [10]. Despite the current lack of consensus on the inclusion/exclusion of the time factor in defining sustainability, the duration covered by the intervention could be used as an index to determine the likelihood of its continuity or sustainability beyond the proposed donor-funded lifecycle. Likewise, the difference between the periods of formal program completion and evaluation of the program results among the beneficiaries could provide information on the effectiveness and sustainability of the intervention.

Africa is described as the poorest region in the World, with Sub-Saharan Africa accounting for only a marginal 3% of the World’s health expenditure [11,12]. With poor funding, the poor state of the health system hinders the attainment of the sustainable development goals (SDGs). To facilitate global attainment of the SDGs, billions of donor funds need to be perennially pumped to improve the health system of African continents. To this end, there is no gainsaying that donor funds have had notable strides in scaling up developmental trajectories in healthcare delivery and health system performance in Africa over the years.

Several issues such as utilization of donor-funded health projects, the impact of such projects, and sustainability of donor support are yet of critical concern. Estimates from the World Health Organization macroeconomics commission revealed that US $2.23 billion (6.25%) of the US $35.53 billion total health expenditure in the World Health Organization African region was from donor sources [13,14]. Further analysis of the estimates showed that only 18 countries received nearly 10% of their total health expenditure from donors, nine nations received an equivalent of 11-20%, seven countries received 21-30% support, six countries got 31-40% aid, while other countries received between 41% and 60% of their health expenditure from donors [13,14]. In 2015 however, it was found that Africa accounted for only 1% of the global health expenditures despite carrying 23% of the global burden of disease [15]. In 2016, health expenditure per capita averaged 80 USD in Africa compared to 4,003 USD in the Organization for Economic Co-operation and Development countries [16,17]. Thus, the healthcare system is characterized by poor political commitment, out-of-pocket payments, and over dependence on donor funding [18,19]. Sequel to the recognition of the positive effects of healthcare expenditure to economic development, African governments made a resolve in 2001 to earmark at least 15% of the annual national budget for the health sector [20]. Nearly 20 years after the resolution, many countries are yet to meet this target. As confirmed by other studies, a comparative analysis on the effects of health expenditure on economic growth between countries in the economic community for the Central African states subregion confirms that health expenditure is a flow not a stock in the path of achieving improved life expectancy and economic development [21-25]. Thus, financial support for the leading diseases of public health importance (HIV, malaria, and tuberculosis) can only be sourced from donors in line with the latter’s priority.

With a rising prevalence of non-communicable and communicable diseases on the African continent, the need for donor funding of health interventions cannot be overemphasized [26-28]. As funding runs out, an evaluation of the program activities is made regarding its success or failure, and factors associated with the failure or success of the program. To derive maximum benefits from significant investment in program development and promote long-term program sustainability, a detailed understanding of the facilitators and barriers of donor-funded projects is important even long after the cessation of funding from primary donors. A study in this regard will be needful to understand the nitty-gritty of project continuity beyond the donor funding lifecycle, as well as help to ensure that the facilitators of program sustainability are activated early enough. This systematic review therefore aimed to determine if implemented donor-funded health related-programs in Africa were sustained beyond the funding lifecycle and determine their facilitators and impeders.

## Review

Methods

Study Design

A systematic review is a research type that presents a rich and critically synthesized body of literature to provide evidence-based knowledge on a subject matter under investigation [29,30]. Building on a systematic review conducted by Iwelunmor et al., we employed a systematic synthesis to expand current knowledge to consider the sustainability of donor-funded health interventions in Africa [31]. Both authors had gained expertise both in the academics and implementation science, and thus constituted the research team. The systematic review was initiated in December 2021 and completed in January 2022.

Population, Intervention, Comparator, and Outcome Elements

The population referred to community members in any country in Africa, the intervention referred to donor funding of health programs/projects, the comparator was the lack of donor funds for health programs/projects, and the outcome in this study was the sustainability of health program/project beyond the donor funding lifecycle.

Search Strategy

A database search was done across three databases namely Google Scholar, PubMed, and Medline principally because many journals are indexed on them. The search terms used in the strategy included "Africa or Sub-Saharan Africa or Central Africa or North Africa or East Africa or West Africa or Southern Africa or Algeria or Angola or Benin or Botswana or Burkina Faso or Burundi or Cape Verde or Cameroon or Central African Republic or Chad or Congo or Cote d’Ivoire or Comoros or Democratic Republic of the Congo or Djibouti or Equatorial Guinea or Eritrea or Eswatini or Ethiopia or Gabon or Gambia or Ghana or Guinea or Guinea-Bissau or Kenya or Ivory Coast or Lesotho or Liberia or Libya or Mali or Madagascar or Malawi or Mauritania or Mauritius or Mozambique or Namibia or Niger or Nigeria or Rwanda or Sao Tome & Principe or Senegal or Seychelles or Sierra Leone or Somalia or South Africa or Sudan or South Sudan or Swaziland or Tanzania or Togo or Tunisia or Uganda or Zambia or Zimbabwe" and "sustainability or capacity building or capacity improvement or sustenance or sustainable or continuity" and "Health interventions or health system or intervention studies or intervention implementation or evidence-based science or evidence-based medicine." A major focus was placed on the term “sustainability” due to its suitability to capture the lived experiences in the continuity of sponsored projects without introducing a broader range associated with the use of similar concepts such as “institutionalization” or “routinization” [31,32].

We modeled our search strategy by drawing on lessons learned from a previously conducted systematic review and thus incorporated only studies pertaining to the current state of health interventions on the continent [31]. Furthermore, a search of the reference list of included studies was assessed for retrieval of eligible articles.

Inclusion and Exclusion Criteria

This study had two inclusion criteria. All peer-reviewed articles on sustainability of health interventions in Africa published between 2015 and 2021. The timeframe for the review was due to the existing gap in knowledge on the sustainability of donor-funded health interventions in Africa and associated factors during the period. All studies that provided one or more context-relevant definitions of sustainability were also included. On the one hand, the Sustainability Framework emphasizes the intervention, the context of its delivery, and the broader environment within which health and healthcare systems operate. On the other hand, Shediac-Rizkallah and Bone’s framework conceptualizes sustainability using elements such as features of the project, organizational factors, and community-related factors.

The exclusion criteria were as follows: (i) publications with no use of quantitative or qualitative methods, including policy briefs, letters, and commentaries; (ii) review articles; and (iii) studies with no information on project evaluation after initial implementation.

Data Extraction

The protocol for this systematic review was documented in the International Prospective Register of Systematic Reviews (PROSPERO) [33]. Screening of titles and abstracts was done, and the full texts of all relevant articles were retrieved. Ilesanmi﻿ OS and Afolabi AA independently assessed the full texts for eligibility and extracted data on the study design, type of intervention, and findings of each article. In events where both authors could not reach a conclusion regarding the inclusion or exclusion of an article, a third party was consulted. The risk of bias in systematic reviews (ROBIS) tool was used to assess the risk of bias in the systematic review [34]. The ROBIS tool is composed of two phases. Phase one was used to assess the relevance of the review, while phase two was used to identify concerns with the review process. Phase two had four domains, namely: study eligibility criteria, identification and selection of studies, data collection and study appraisal, and synthesis of findings. Responses to each question were graded from “yes” to “probably yes,” “probably no,” “no,” and “not indicated.” The risk of bias assessment in each domain was summarized into three categories: “high risk,” “low risk,” and “unclear.”

Results

**Figure 1:**
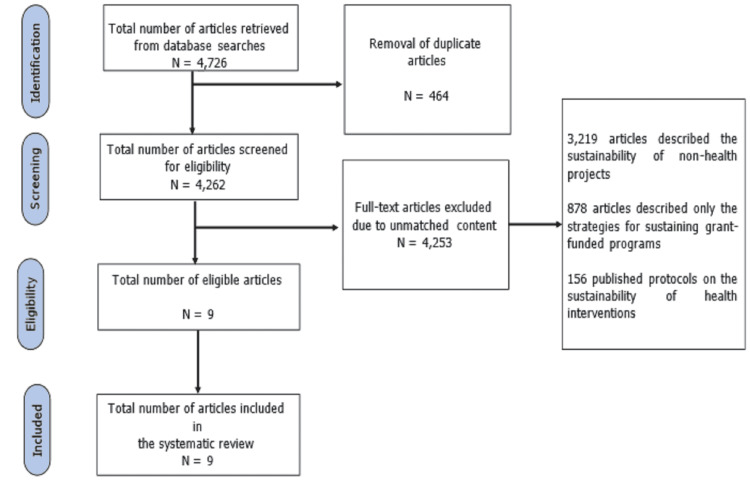
PRISMA flowchart showing the article search strategy. PRISMA: Preferred Reporting Items for Systematic Reviews and Meta-Analyses

The flow chart of the search strategy is presented using the Preferred Reporting Items for Systematic Reviews and Meta-Analyses (PRISMA) framework (Figure 1) [35]. Overall, 4,876 articles were retrieved, and only nine articles were eligible for inclusion in the review following the removal of duplicates and application of the inclusion and exclusion criteria.

Scope Covered by the Study

Only three of the five regions in Africa were represented, with three (33.3%) from West Africa, two (22.2%) from East Africa, three (33.3%) from Southern Africa, and one (11.1%) conducted in East Africa and Southern Africa. Overall, 10 countries were represented in this review with Nigeria having the highest number of literature included. Three (33.3%) articles were published in 2015, two (22.2%) were published in 2017, two (22.2%) were published in 2018, and two (22.2%) were published in 2019.

Framework/Theory and Methodologies Used

All (100%) of the articles included in the review discussed sustainability using a framework or theory. Among them, six (66.7%) utilized the sustainability framework, only two (25.0%) used the systems thinking-guided framework, and one (12.5%) utilized both the sustainability framework and theory of change.

Majority of studies reviewed utilized qualitative methods, including in-depth interviews, focus group discussions, and key informant interviews (n=5). Some studies also included the use of rapid desk review (n=1) or traditional sources of data such as population census (n=1). Mixed methods comprised one-quarter of included articles (n=3).

Timeframe of Assessment

Coding of studies for the post-implementation report obtained was done. Among them, three (33.3%) were assessed within two years post-implementation, one (12.5%) was assessed one-year post-implementation, and one (12.5%) was assessed more than five years after implementation. In four (50.0%) studies, the period of execution of the project and/or assessment was not stated.

**Figure 2:**
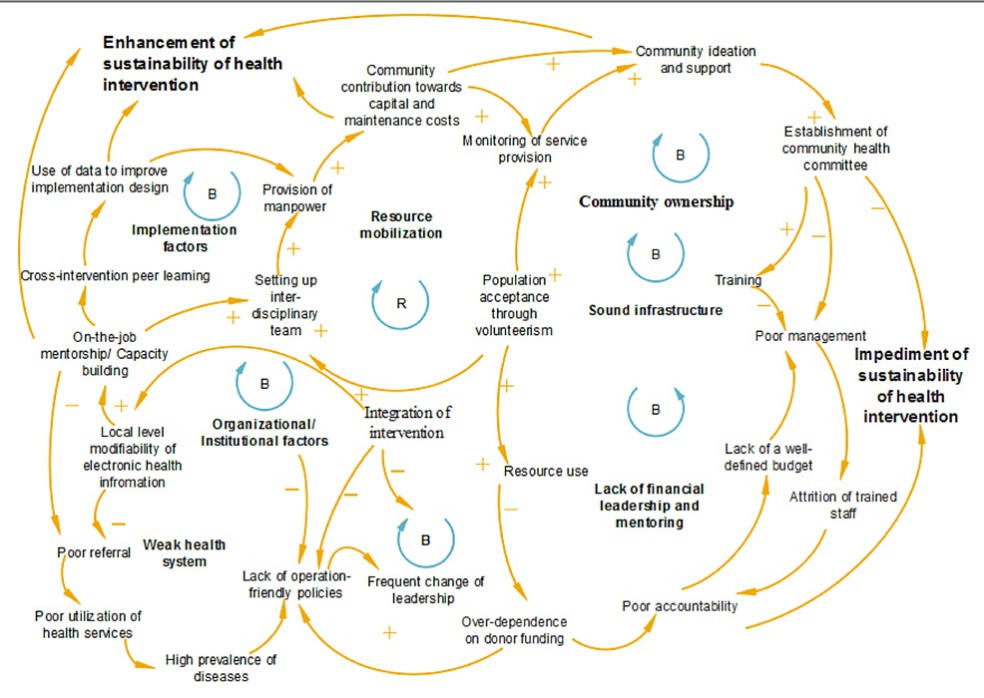
Causal loop diagram of the mechanism of interaction of sustainability of health interventions between the community, health system, and the sociopolitical context. The diagram is developed and designed by the authors of this study.

Table 1 summarizes the findings of literature included in the review, while Table 2 summarizes the facilitators and impeders that influenced donor-funded health program sustainability. Figure 2 shows the causal loop diagram of the mechanism of interaction between the community, health system, and the sociopolitical context.

**Table 1:** Summary of literature included in the systematic review.

S.n.	Source literature (country)	Theory/framework	Intervention	Definition of sustainability	Analytical method	Timeline of project (period of assessment)	Project was sustained beyond the funding life cycle?	Results/implications
1	Mutale et al., 2015 (Zambia)[36]	Systems thinking-guided analysis framework	BHOMA intervention (better health outcome through mentorship and assessment)	Improvements in service quality leading to increased demand for health services from the community	In-depth interview (for health workers and community representatives) and focus group discussion (for community members)	2011-2014 (2014)	Yes	Improvements in patient triaging system, record keeping, health worker mentoring and training, and comprehensive consultation
2	Kiwanuka et al., 2015 (Eastern Uganda: Pallisa and Kamuli LGAs)[37]	Sustainability framework	Rural water and sanitation (RUWASA) projects	(i) Promotion of access to safe water using technology. (ii) Reduction in the prevalence of water-borne diseases	Key informant interviews (for program managers and officers) and Focus group discussion (for community members)	1997-1999 (in Kamuli LGA) and 1995-2001 (in Pallisa LGA) (Period of assessment: March 2012)	Yes	(A) Drilling of boreholes: in Pallisa LGA: at the start of the project in 1997, Pallisa had 9 boreholes drilled, by the end of the project in 2001, they reached a total of 21, which kept increasing steadily to a total of 320 in 2011. In Kamuli, they started with 421 boreholes drilled, 557 at the end of the project in 1999 and reached 1,023 in 2011. (B) Safe water coverage: this increased by 7% and 6% in Pallisa and Kamuli respectively. However, by 2011 both LGAs both exceeded 65%. (C) Latrine coverage improved over the years with both LGAs starting at 7% in 1997, 24% when the project ended (in 2001), and >65% at the end of 2011
3	Ibrahim and Wan-Puteh, 2018 (Nigeria)[38]	Sustainability framework		Sufficient community engagement at the beginning of a project, middle, end, and after	Key informant interviews (civil society advisors, heads of departments, program officers, trustees, and executive board members)	Not stated	Yes	Partnership enhances project sustainability
4	Moucheraud et al., 2017 (Malawi, Zambia, and Zimbabwe) [39]	Sustainability framework	Electronic health information system (EHIS) projects funded by U.S. President’s Emergency Plan for AIDS Relief via the Centers for Disease Control and Prevention	The capacity to maintain continuous running of programs and services after the end of financial, managerial, and technical assistance from external donors.	Interviews (employees at government ministries, clinical and data/clerical staff at health facilities, and those involved “upstream” in the EHIS (software developers, managers, advisors, and board members)	The project started in 2001 in Malawi, 2009 in Zambia, and 2009 in Zimbabwe (period of assessment was not stated)	Yes	The engagement of users in the design and implementation process is needful to achieve sustainability
5	Rwabukwis et al., 2017 (Ghana, Mozambique, Rwanda, Tanzania, and Zambia)[40]	Systems thinking-guided analysis framework	Population Health Implementation and Training partnership projects funded through African Health Initiative funded by the Doris Duke Charitable Foundation (DCCF)	Health system capacity after the termination of donor funding for the projects	Key informant interviews (implementation leaders); rapid desk review of available program documents and annual and six-month reports submitted to DDCF; semi-structured interviews (at least one participant from each country team)	Period of projects was not stated (October 2015)	Yes	A substantial improvement in health indicators amidst sustained fundamental challenges to health systems could only be achieved through excellent stakeholder/community engagement
6	Onwejekwe et al., 2019 (Nigeria)[41]	Sustainability framework	Free maternal and child health program by the National Health Insurance Scheme (NHIS) using funds from the debt relief gains (12 states in Nigeria)	Provision of adequate and sustainable funding in a predictable and regular manner to reduce disruptions in service delivery	In-depth interviews with key informants (officers of the NHIS, Office of the Senior Special Assistant to the President on the Millennium Development Goals, Health Maintenance Organizations, Public health facilities, state/local government, as well as community members Desk review (policy documents, program implementation reports, and other relevant reports)	2009-2015 (February to August 2016)	No	States are required to explore innovative financing strategies to increase the possibility of sustaining free maternal and child health program
7	Kilewo and Frumence, 2015 (Tanzania) [42]	Sustainability framework	Comprehensive council health plans	Improved operationalization of community engagement efforts	In-depth interviews with key informants from health facility governing committees, council health service board, and council health management team	Not stated (May 2013)	No	Decentralization by devolution policy should be ensured so that local governance structures have adequate resources for the planning and management of planning and managing comprehensive council health plans
8	Speizer et al., 2019 (Nigeria) [43]	Sustainability framework/ theory of change	Demand generation activities	Improvement in FP access and contraceptive use in urban areas through comprehensive demand and supply-side programming	Population census; quantitative survey from community members	2009-2014 (phase 1: 2015; and phase 2: 2017)	Yes	Program effects sustained for up to two years out and were strongly correlated to ideation
9	Oldewage-Theron et al., 2018 (South Africa)[44]	Dynamic Sustainability framework	Improving household food security in Free State and Gauteng project	(i) Best practices to improve health and wellbeing. (ii) Improvement in household food insecurity	Key informant interviews and desk reviews (of photographs, and journal logs)	2011-2013 (2013)	Yes	Program was sustained after funding ended, and was strongly correlated to the inclusion of community stakeholders in the supervisory team for the project

**Table 2:** Summary of facilitators and impeders of sustainability of health intervention projects in Africa.

Facilitators	Examples
Community ownership	(i) Commitment of district managers [36]. (ii) Engagement of district officials and community members in needs assessment and priority setting exercises [37,41]. (iii) Traditional leadership involvement in rural areas [37]. (iv) Establishment of community water development committees that ensured appropriate use of funds [38]. (v) Social contracting [38]. (vi) Community receptivity to electronic health information system [39]. (vii) Community ideation and support [43,44]. (viii) Availability of contracts of clinic supporters to reduce health workers’ workload [36]
Working within existing resources	(i) Integration of intervention within existing political structures [36,40]. (ii) Availability of rural drug kits in all community-based health facilities [36]. (iii) Integration of electronic health information management system into the national and local health system [39]. (iv) Strengthening of local policies and management structure [40]. (v) Efficiency in the use of public resources to provide access to critical interventions to vulnerable populations [41]. (vi) Direct transfer of funds to health facilities [41]
Sound infrastructure	(i) Community participation in developing work plans for operating and maintaining the infrastructure [41]. (ii) Training of community members on the repair and maintenance of equipment [37]. (iii) Opportunities for improved computer literacy [39]
Resource mobilization	(i) Community members’ provision of manpower and contribution towards capital and maintenance costs[36]. (ii) Mobilization of resources for effective functioning [38]
Organizational/contextual factors	(i) Local-level modifiability of electronic health information system and extensive engagement of local partners [39]. (ii) Interdisciplinary team (National and International staff, and expertise in local context) [40]. (iii) On-the-job mentorship and capacity building [40]
Implementation factors	(i) Cross-intervention peer learning [40]. (ii) Use of data to inform iterations on intervention and implementation design [40]
Impeders	Examples
Weak health systems	(i) Poor filing systems [36]. (ii) Unreliable referral services [36]. (iii) Poor integration of electronic records into the national health management information system [36]
Lack of financial leadership and mentoring	(i) Lack of health workers’ integrity (misuse of drugs and supplies) [36]. (ii) Poor accountability [36,41]. (iii) Lack of structured training, monitoring, and supervision [36,41,42]. (iv) Poor remuneration for clinic supporters [36]. (v) Dependency on donor funding [38,41] vi. Lack of well-defined budget [42]
Shortage of efficient health resources	(i) Non-availability of clinic supporters during night and weekend shifts [36,40]. (ii) Non-engagement of local health workers such as traditional birth attendants and community health workers [36]. (iii) Poorly trained new health managers [37]. (iv) High attrition of trained staff [36,40]. (v) Lack of resources (human, money, and machineries) [38,40,41]
Organizational and institutional factors	(i) Separation of the water component from the sanitation component by the Ministry of Health [37]. (ii) Separation of electronic health information system from other reporting systems [39]. (iii) High cost and relative scarcity of software development and technical support [39]. (iv) Frequent change of project leadership; and staff turnover necessitating constant training [40]. (v) Non-assignment of roles and responsibilities [42]
Contextual/socio-political factors	(i) Political interference [36,39,40]. (ii) Weak donor-community interactions [39]. (iii) Identification of the ideal moment for active engagement of users of the intervention [39]. (iv) Lack of community involvement in the design of the project [41]. (v) A lack of operation-friendly policies [38]. (vi) High prevalence of diseases [40]
Poor feedback mechanism	Poor communication and information sharing between Council Health Management Team and Health Facility Governing Committees [42]
Implementation factors	(i) Local intermittent change in data tools and methods [40]. (ii) Funding challenges and poor service utilization [41]. (iii) Non-involvement of the community in the implementation of the project [42]. (iv) Lack of sustainability plans [43]

Discussion

This systematic review aimed to determine if implemented donor-funded health interventions were sustained beyond the funding lifecycle, as well as the facilitators and impeders of the sustainability of such interventions. This study identified that more than six donor-funded projects were sustained beyond the funding lifecycle from donor organizations. Despite the poor socioeconomic status in which most of the beneficiary communities existed, it could be inferred that communities mobilized internal support to sustain donor-funded health interventions [45-47]. This finding thus elucidates that the sustainability of health interventions does not rest solely on the oars of provisions from donors [48]. A study conducted to assess the rural water and sanitation projects in Pallisa and Kamuli LGAs in Eastern Uganda (one of the studies included in the review) reported that drilled boreholes increased by more than 10 folds in Pallisa and by nearly two folds in Kamuli about 10 years after donor funds had been withdrawn [37]. The huge difference in latrine coverage between the formal period of project completion (24%) and sustainability assessment (>65%) could have only been achieved because community members identified the huge benefits of the project and thus sustained its maintenance [37]. Similar findings were recorded by Mutale et al. of the BHOMA intervention in Zambia where improvements in the quality of health service provision led to an increased demand for health services from community members [36].

The sustainability of the electronic health information system projects funded by the President’s Emergency Plan for AIDS Relief in Malawi, Zambia, and Zimbabwe likewise gained continuity beyond the period of funding by donors [40]. In a qualitative evaluation of implementation experience across five African countries, Population Health Implementation and Training project leaders and their teams had to respond to different contexts that arose right from the start and throughout the lifespan of the project [40]. Experience from applying the systems thinking approach shows that public health interventions require a comprehensive approach as well as a strong and efficient implementation framework [36,40,49]. The results from this systematic review underscore the importance of creating an enabling environment for sustaining health interventions by aligning perspectives and engaging users [50]. It is also beneficial to take an extensive view of sustainability that considers financial dimensions as well as other determinants of program sustainability. Sustainability is a resource-intense endeavor that is particularly beneficial in low-resourced settings such as Africa, and it is essential to ensure that facilitators of program sustainability are enhanced while impeders are addressed.

Findings from this study revealed that community ownership of donor-funded projects through the commitment of district representatives, and involvement of traditional leaders is a reliable strategy towards achieving sustainability of health interventions. It could be deduced that community members and stakeholders had adequate knowledge of each project and their role in ensuring its continuity. As a result, it was easy to implement the project within existing community resources including material, financial, and human. This result is similar to the findings from other studies where it was reported that the uncertainty of community stakeholders about their roles and responsibilities in the execution of donor-funded projects resulted in poor performance of the assigned intervention [41,42,44]. Based on this knowledge, clarity of roles and responsibilities has been emphasized by the United Republic of Tanzania as a critical factor that strengthens autonomy of health board on any donor-funded project [51].

This study found that community acceptance of a health intervention through volunteerism is a core factor that drives the sustainability of any donor-funded project. This is because community engagement strategies are adopted at the onset of the project using a grassroots design that helps to create a sense of ownership and minimize dependency on the funding agencies at the community level [45]. As revealed in some studies included in this review, the intellectual, human, and material resources of the community are tapped into with the recognition that these resources are crucial to ensure the project of the success through the grassroots approach [36-38]. Because community acceptance of a project fosters indigenous democratic elements and civil society development that reflect local values, community members and stakeholders through their organized groups and sound infrastructure can continue to drive a project long after financial assistance from the donor is withdrawn [36,38,39].

From this study, we identified the roles played by organizational and implementation factors in ensuring the achievement of a project’s objectives and ensuring sustainability. Adequate engagement of local partners and inter-disciplinary team with expertise, and satisfactory use of data to promote cross-intervention peer learning were identified from the included literature as facilitators of project sustainability [39]. From the Water and Sanitation program in Indonesia, local communities had water supply because the community-based water supply management board was empowered as community forum [52]. Support from local personnel always helps towards developing water supply management boards so that local communities have a reliable water supply system managed by the community itself.

This study also provided insight into certain factors that could serve as a barrier to the sustainability of health intervention projects. Due to poor documentation using electronic records, any details on the progress of the report go missing [36]. Consequently, these further weaken the already weak health system. In many local communities especially in developing countries, failure to engage unskilled health workers such as traditional birth attendants (often called doulas) and community health workers could serve as a clog in the wheel of progress to the sustainability of health intervention projects [36,40]. Although they are unskilled, traditional birth attendants and community health workers could be potential human resources that can motivate community-wide acceptance of the project. Due to their excellent understanding of the terrain, they could provide rich information on community entry and community engagement strategies. Due to the shortage of skilled health human resources, local health workers could be readily trained as personnel and thus offsetting extra funds to remunerate newly engaged staff.

Broader sociopolitical factors are external impeders that could obstruct the continuity of a donor-funded project beyond the funding lifecycle. According to the World Health Organization building block of the health system, management or political factors could pose the greatest threat to the sustenance of a funded health intervention [40,41,53,54]. With the lack of operation-friendly policies, the funded project may lose priority before health workers and community members who were originally intended to benefit from the project [36]. If the beneficiaries of an intervention consider the intervention of no huge benefit to them, how then do we expect them to ensure that the project does not fail? Thus, the failure of the sociopolitical factors to provide context-based support could influence a lack of financial leadership, mentoring, and accountability. If funds are insufficient to drive the intervention, volunteer health workers are likely to be poorly remunerated. There is also a likelihood that health workers’ integrity would be lacking since drugs and supplies would be used for personal benefits only to augment the poor remuneration.

Limitations

This study was limited to the facilitators and impeders of sustainability of donor-funded health interventions in Africa. Given the context, our results may not be generalizable to some low-income countries that are supported by donor funds.

## Conclusions

Donor-funded health and health-related projects are highly beneficial in the restoration, and preservation of community health, and could be sustained by factors such as community ownership and acceptance through volunteerism, working within existing resources, and training. Despite the benefits of donor-funded projects, factors such as weak health system, lack of financial leadership and mentoring, and shortage of efficient health resources could prevent the continuity of funded health interventions. Therefore, it becomes needful that identified facilitators of sustainability are promoted, while the impeders are immediately addressed through some strategies. Firstly, adequate engagement of local (unskilled) community health workers should be done, as it would be required to enable local stakeholder buy-in and enhance community acceptance of the intervention. Secondly, good reporting system, as well as the integration of paper-based records onto a centralized electronic record, should be instituted to prevent loss of critical information that could be beneficial to the sustenance of the intervention. In addition, operation-friendly policies should be established, and certain funds should be allocated on a steady basis from the budget of each local health management authority to provide some financial support to communities where funded projects are ongoing.
